# HOMER: a human organ-specific molecular electronic repository

**DOI:** 10.1186/1471-2105-12-S10-S4

**Published:** 2011-10-18

**Authors:** Fan Zhang, Jake Y Chen

**Affiliations:** 1 School of Informatics, Indiana University, Indianapolis, IN 46202, USA; 2 Department of Computer and Information Science, School of Science, Purdue University, Indianapolis, IN 46202, USA; 3 Indiana Center for Systems Biology and Personalized Medicine, Indianapolis, IN 46202, USA

## Abstract

**Background:**

Each organ has a specific function in the body. “Organ-specificity” refers to differential expressions of the same gene across different organs. An organ-specific gene/protein is defined as a gene/protein whose expression is significantly elevated in a specific human organ. An “organ-specific marker” is defined as an organ-specific gene/protein that is also implicated in human diseases related to the organ. Previous studies have shown that identifying specificity for the organ in which a gene or protein is significantly differentially expressed, can lead to discovery of its function. Most currently available resources for organ-specific genes/proteins either allow users to access tissue-specific expression over a limited range of organs, or do not contain disease information such as disease-organ relationship and disease-gene relationship.

**Results:**

We designed an integrated Human Organ-specific Molecular Electronic Repository (HOMER, http://bio.informatics.iupui.edu/homer), defining human organ-specific genes/proteins, based on five criteria: 1) comprehensive organ coverage; 2) gene/protein to disease association; 3) disease-organ association; 4) quantification of organ-specificity; and 5) cross-linking of multiple available data sources.

HOMER is a comprehensive database covering about 22,598 proteins, 52 organs, and 4,290 diseases integrated and filtered from organ-specific proteins/genes and disease databases like dbEST, TiSGeD, HPA, CTD, and Disease Ontology. The database has a Web-based user interface that allows users to find organ-specific genes/proteins by gene, protein, organ or disease, to explore the histogram of an organ-specific gene/protein, and to identify disease-related organ-specific genes by browsing the disease data online.

Moreover, the quality of the database was validated with comparison to other known databases and two case studies: 1) an association analysis of organ-specific genes with disease and 2) a gene set enrichment analysis of organ-specific gene expression data.

**Conclusions:**

HOMER is a new resource for analyzing, identifying, and characterizing organ-specific molecules in association with disease-organ and disease-gene relationships. The statistical method we developed for organ-specific gene identification can be applied to other organism. The current HOMER database can successfully answer a variety of questions related to organ specificity in human diseases and can help researchers in discovering and characterizing organ-specific genes/proteins with disease relevance.

## Background

Organ-specific patterns of gene expression can give important clues about gene function and organ characteristics. High-throughput sequencing methods offer the opportunity to examine patterns of gene expression on a genome scale and generate an abundance of data describing the expression of gene transcripts within various human organs and disease states to facilitate transcriptomic studies [1]. Organ-specificity expression profiling has been widely used for identifying potentially therapeutic genes related to specific organs [2] and understanding the characteristics of cells and tissues in an organ in terms of their differential expression of genes [3]. For example, Andrew Su etc. have designed custom arrays that interrogate the expression of the vast majority of protein-encoding human and mouse genes, and have used them to profile a panel of 79 human and 61 mouse tissues or organs [4]. Previous researches have identified organ-specific genes that are specifically expressed in the testis [2], the heart [5], the prostate [6], the brain [7], and the bladder [8] etc. For example, Kouame etc. identified the genes uniquely detected in each of the 15 tissues or organs such as testis, prostate, ovary, mammary gland, uterus, vagina, skin, liver, adipose tissue, lung, bone, skeletal muscle, cerebral cortex, hypothalamus, and pituitary gland. Their study shows that 61 organ-specific transcripts in the testis are statistically different from the other organs and that some transcripts such as dipeptidase 3, ankyrin repeat domain 5, and ubiquitin-conjugating enzyme E2N are exclusively found in the testis [2]. They have also identified some prostate specific genes such as microseminoprotein (beta-MSP), seminal vesicle protein secretion 2, seminal vesicle antigen (SVA) and mucin 10 (MUC10) which are involved in protein secretion, cell signaling and spermatogenesis.

For “organ-specificity of gene expression”, we refer to differential expressions of the same gene across different organs. In particular, we define an “organ-specific gene/protein” as a gene/protein whose expression is significantly elevated in a specific human organ. However, the expression level of the organ-specific gene/protein may vary in an organ under certain circumstances, which makes the organ-specificity questionable. Therefore, we need to quantify organ specificity based on organ context. Highly expressed genes/proteins with high quantitative organ specificity levels are also implicated in human diseases related to the organ. In other words, they may be used as an indicator of the normal/abnormal physiological states of the organ. We refer to them as “organ-specific markers”.

The organ-specific gene/protein can be used as an indicator to measure the function of a tissue in a respective organ. The organ-specific gene/protein can indicate important clues about gene function [4] and also monitor organ integrity both during preclinical toxicological assessment and clinical safety testing of investigational drugs. Additionally, it may provide valuable information for decision making during toxicological assessment and may be used for sensitive and specific target organ monitoring during clinical trials [9].

There are a number of databases today that include information on tissue specific expression of genes/proteins, for example, TiGER [10], TiSGeD [11], and HPA [12]. These resources have several limitations. First, they all uses organ name to present tissue. For example, brain is considered as a tissue and not an organ. Tissue is a group of cells that perform specific functions. An organ is a group of tissues that perform a specific function or group of functions. Also it is common to know what organ system is involved in a disease and diseases are mostly categorized by human organ system. Therefore, we need to map tissues to organs and use organ name instead of tissue name for calculating organ-specificity and building the disease-organ association which is more accurate than disease-tissue relationship. Second, they have a low coverage of organs and genes. For example, TiGER [10] covers only 30 organs. It includes expression values for genes and has Gene ID’s, but no protein information is presented. 1,494 out of 6,698 UniGene IDs have been retired since its last update in 2008. In TiSGed [11], 18 organs are covered. It defines tissues by organ name in a tree fashion, but all tissues in an organ are not covered and protein information is not presented. HPA (Human Protein Atlas) [12] provides a range of 74 tissue-specific proteins which cover 24 organs based on the protein levels in 65 normal cell types. Although HPA’s normal tissue data contains 11261 Ensembl genes, their expression values are based on the annotated expression levels: “Negative”, “Moderate”, “Strong”, “Weak”, “Medium”, “High”, “None”, and “Low.” No real number value for expression is given, which makes digitizing the expression values very challenging and calculating organ specificity questionable. For example, How to accurately digitally distinguish between the “Strong” and “High”, the “Weak” and “Low”, and the “Moderate” and “Medium?”. Last, they don’t contain disease information such as disease-organ relationship and disease-gene relationship.

For studies focusing on organ-specificity with relation to diseases, it is desirable that the database should house data from a range of organs, have quantitative organ specificity and, more importantly, disease information. Therefore, as described in this paper, we designed an integrated database defining human organ-specific molecule (gene/protein). In our organ-specific molecule design we considered five criteria: 1) comprehensive organ coverage; 2) gene/protein to disease association; 3) disease-organ association; 4) quantification of organ-specificity; and 5) cross-linking of multiple available data sources.

The Human Organ-specific Molecular Electronic Repository (HOMER), located at http://bio.informatics.iupui.edu/homer/ is a comprehensive database covering about 22,598 proteins, 52 organs, and 4,290 diseases integrated from databases including dbEST [13], TiSGeD [11], HPA [12], CTD [14], and Disease Ontology [15]. It is the first comprehensive database that can be used to analyze, identify, and characterize organ-specific molecules in association with disease-organ and disease-protein relationships. The gene/protein to disease and disease-organ associations allow future identification of organ-specific markers. The comprehensive 52 organs in 13 human organ systems and the ability to choose quantitative variables (p-value, z-score, #EST, and Adjusted #EST) provide us with power statistics and computation to accurately calculate organ specificity. And the cross-linking of multiple data sources enables subsequent validation.

The database has a Web-based user interface that allows users to query organ-specific genes/proteins by gene, protein, organ, or disease, browse organ-specific genes/proteins by human organ system and disease ontology, explore a histogram of each organ-specific gene/protein, and identify disease-related genes or disease-related organs.

Moreover, two case studies were performed to demonstrate and validate that the repository can help researchers discover and characterize organ-specific protein molecules implicated in human diseases related to the organ: 1) an association analysis of organ-specific genes with disease and 2) a gene set enrichment analysis of organ-specific gene expression data.

## Results

### Database content statistics

By integrating organ-specific protein/genes and disease databases including dbEST [13], TiSGeD [11], HPA [12], CTD [14], and Disease Ontology [15], we have developed HOMER, the Human Organ-specific Molecular Electronic Repository. As of the current release (June 2011), HOMER contains 22,598 proteins (IPI IDs), 5,703 genes (gene IDs), 52 organs, and 4,290 diseases (MeSH IDs) of which 4492 are disease-related organ-specific genes (gene IDs) and 2000 are identified as organ-specific markers (gene IDs) (Table 1). A comparison of organ-specific genes/proteins in HOMER against several common human tissue/organ-specific data sources is shown in Table 2.

**Table 1:** Current statistics of database

Total Number	Count
Organs	52
Genes	111367 UniGene IDs
Proteins	76755 IPI IDs
Organ-specific Genes	5703 GeneIDs, 6999 UniGeneIDs
Disease-related and Organ-specific Genes	4492
Organ-specific Markers	2000
Diseases	4290

**Table 2:** A comparison of human organ-specific genes/proteins in HOMER against several common human tissue/organ-specific data sources

	**TissueDistributionDBs**[3]	**TiGER**[10]	**TiSGeD**[11]	**HPA**[12]	HOMER
Organ coverage	40	30	18	24	52

Gene coverage	1359*	4283**	957***	74	5703

Last Updated	2010	March 2008	July 2010	Jan 2011	June 2011

Criteria for organ-specific genes	TissueSpecificityIndex	p-value<1e-3.5 EE>5	SPM	Exclusively detected in a single cell	p-value ≤ 1e-5 *RZ* ≥ 4 *AE* ≥ 10 *RE* ≥ 4

Expression Value	Relative expression	Yes	No	No	Relative Expression

Plasma Detectability	No	No	No	No	Yes

Disease Association	No	No	No	No	Yes

*1359 gene IDs were filtered out from 54, 576 human UniGene IDs with TissueSpecificityIndex = 1. We used TissueSpecificityIndex = 1 as the website didn’t recommend any criteria for us to derive organ-specific genes.

** 1494 of 6698 UniGene IDs were retired.

*** 957 gene IDs were filtered out from 2423 gene IDs with SPM ≥ 0.9. We used SPM ≥ 0.9 as the website didn’t recommend any criteria for us to derive organ-specific genes.

### General online features

**Figure 1:**
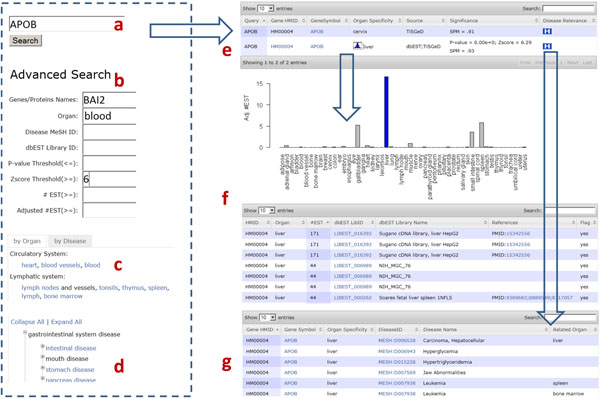
**Web interface structure.** a) Query organ-specific genes by genes or proteins. For example, a UniGene ID, an Entrez gene ID, a gene name, a uniprot ID or IPI ID are all supported. To enter multiple values, delimit them by comma, semi-colon or space. b) advanced search. Query in customized ways, including referencing a list of genes/proteins, searching within p-value, z-score, number of EST, and adjusted number of EST ranges, or looking for organ-specific genes/proteins related to specific organ, disease MeSH ID, or dbEST library ID. c) browse organ-specific genes/organs by human organ system. d) browse organ-specific genes/organs by disease ontology. e) search result. In the gene/protein organ specificity table, it shows gene HMID, gene symbol, organ specificity, source, significance (p-value and z-score), and disease relevance. Users can further explore the histogram of the organ-specific gene/protein across the 52 organs by clicking on the histogram icon in the column of organ specificity, and its disease relevance by clicking on the disease relevance icon in the last column. f) histogram of organ-specific gene/protein. g) disease relevance of organ-specific gene/protein.

In Figure 1, we show the user interfaces of the web-based online version of HOMER. It supports both standard and customized search options that allow users to specify a list of genes/proteins or keywords as the query input. In the Advanced Search interface, users can drill down in very specific ways, including referencing a list of genes/proteins, searching within p-value, z-score, number of EST, and adjusted number of EST ranges, and looking for organ-specific genes/proteins related to specific organs, disease MeSH IDs, or dbEST library IDs. One of the more interesting features of HOMER is the ability to browse for organ-specific genes/proteins by human organ system and disease ontology.

In response to these queries, HOMER can retrieve a list of related organ-specific genes in a highly flexible table, with which users can further explore details about organ-specific genes/proteins. For example, users can browse gene symbol, p-value and z-score for each gene/protein, explore the organ-specific expressions of the HMID by clicking on the histogram icon in the table, and look through the gene-related diseases and disease-related organs by clicking on the disease relevance icon in the last column. In the histogram, users can browse the dbEST libraries and reference sources which contain the ESTs related to the gene/protein. The organ-specific genes/proteins are freely available for downloading in tab-delimited format on the download page. User queried organ-specific gene/protein data stored in HOMER can also be freely downloaded as tab-delimited text files using links below each organ-specific gene/protein table.

### Overlap of OSGs among organs

**Figure 2:**
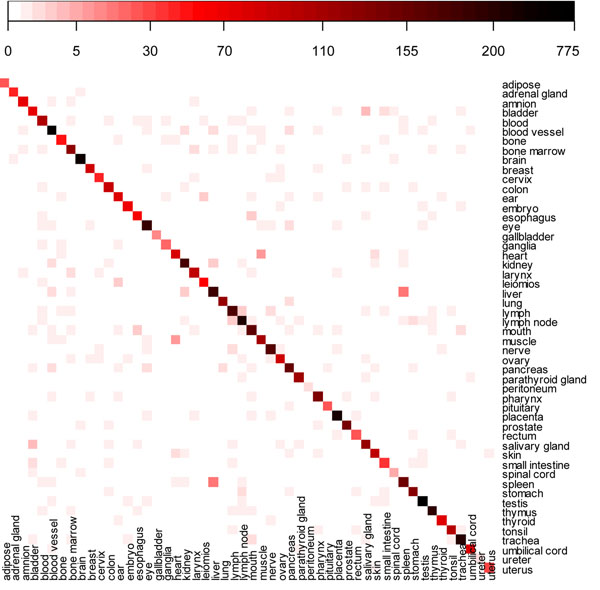
**Heatmap of organ-specific genes among organs.** x-axis and y-axis are both 52 organs. The degrees of redness and blackness in each cell represent increase of number of overlapping genes between organs. The legend above the heatmap indicates the range of number of overlapping genes between organs. It is nonlinear color scale from white to red to black, correspondingly, indicating the value scales from 0 to 775.

We used a heatmap to show the overlap of OSGs among the 52 organs (Figure 2). The 3 organs which show more than 300 organ-specific genes are testis (773); blood vessel (549); and brain (369), while gallbladder (11), spinal cord (6), peritoneum (2), and ureter (2) have the least number of organ-specific genes in our study.

When we tightened the criteria from *RZ* ≥ 4 to RZ ≥ 5, we found that there is no overlapping among the 52 organs. We also found that the distribution of organ specificity of genes between the 52 organs marginally changes with the increase in relative z-score. This suggests that those top organs with more organ-specific genes are much more organ-specific than the other organs.

Figure 2 shows that the liver and the spleen have the largest number of OSGs in common: 16. The other large overlapping of OSGs between organs are heart and muscle (7), bladder and salivary gland (4), ear and leiomios (3, leiomyoma), esophagus and mouth (3), and lymph and lymph node (3).

### Validation by HPA

Selecting the top three genes from each organ, we found 154 organ-specific genes in UniGeneIDs (152 in gene IDs; peritoneum and ureter only have two organ-specific genes, 73 match with HPA data, Additional File 1). Based on expert experience, we digitalized the annotated protein expression in HPA. On a scale of 0 to 9, ‘None’ 0, ‘Negative’ is 1, ‘Low’ 2, ‘Weak’ 3, ‘Medium’ 5, ‘Moderate’ 6, ‘High’ 7, and ‘Strong’ 9. After scoring the annotated protein expression, we used the similar statistics method for the dbEST data to calculate the p-value and z-score for HPA and found 25 (34%) out of the overlapping 73 organ-specific genes in HOMER are specific to the same organ in HPA data (Additional File 1).

### Pathway analysis, gene ontology categorization, and drug target analysis of organ-specific genes/proteins

The pathway-gene association matrix for the 154 organ-specific genes is shown in the Additional File 2. The top two pathways are “Neuroactive ligand-receptor interaction” and “Ribosome.” 15 disease/cancer-related pathways are included in the Additional File 2, which are "Pathways in cancer," "Jak-STAT signaling pathway," "Autoimmune thyroid disease," "PPAR signaling pathway," "Chemokine signaling pathway," "p53 signaling pathway," "Type I diabetes mellitus," "Alzheimer's disease," "Amyotrophic lateral sclerosis (ALS)," "Huntington's disease," "Vibrio cholerae infection," "Epithelial cell signaling in Helicobacter pylori infection," "Small cell lung cancer," "Allograft rejection," and "Graft-versus-host disease."

**Figure 3:**
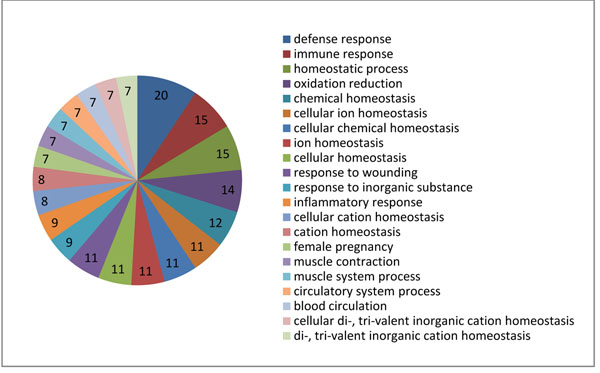
**GO analysis of the 154 organ-specific genes.** The numbers in the pie chart are the number of represented genes in a GO term.

Figure 3 quantifies the significance of the biological process component of the gene ontology. The top 3 biological processes for the 154 organ-specific genes are “defense response,” “immune response,” and “homeostatic process.”

In the Additional File 3, we list all drugs with which those 154 organ-specific genes interact as drug targets. Interestingly, we found some organ-specific drug targets are involved in a particular metabolic or signaling pathway that is specific to the organs as key molecules. For example, the two brain-specific biomarkers SV2A and GRM3 are used as drug targets of Levetiracetam, and Nicotine and Acamprosate, respectively, which is consistent with previous findings. Pediatr etc. studied 23 patients with cancer and seizures treated with Levetiracetam, and they observed that over 95% of patients had fewer seizures, with 65.2% becoming seizure free; only one patient experienced an adverse reaction. They concluded that Levetiracetam is effective and well tolerated in children with brain tumors and other cancers, who are often on multiple enzyme-inducing drugs [16].

One study shows that Nicotine can help improve some of the learning and memory problems associated with hypothyroidism. Such studies suggest that nicotine -- or drugs that mimic nicotine -- may one day prove beneficial in the treatment of neurological disorders [17]. Another new study has found that one of nicotine's metabolites, cotinine, may improve memory and protect brain cells from diseases such as Alzheimer's and Parkinson's [18].

Acamprosate, also known by the brand name Campral, is a drug used for treating alcohol dependence. Acamprosate is thought to stabilize the chemical balance in the brain that would otherwise be disrupted by alcoholism, possibly by blocking glutaminergic N-methyl-D-aspartate receptors, while gamma-aminobutyric acid type A receptors are activated [19].

### Case studies

It has been reported that organ-specific genes are often implicated in diseases related to specific organs. However, it remains largely unknown whether there is a correlation between the organ specificity of a gene/protein and the diseases associated with the organ. We show two case studies of increasing complexity and biological significance to achieve three goals: 1) to demonstrate that the database can help researchers discover and characterize organ-specific genes/proteins from experimental data, 2) to test the hypothesis that there is correlation between the organ specificity of a gene/protein and the associated diseases, and 3) thereby to validate the usefulness of our database.

### Case study 1: website features

The liver is the human body’s one of most important organs, functioning as a living filter to clean the system of toxins, metabolize proteins, control hormonal balance, and produce immune-boosting factors. In this case study, we illustrate the features of HOMER by testing the association between liver-specific genes/proteins and the liver diseases.

We first investigated the liver-specific gene/protein by querying organ by liver (Figure 1b and 1c). We obtained 317 liver-specific genes (195 in dbEST, 193 in TisGeD [11], 2 in HPA). These proteins include major plasma proteins such as ALB, factors in hemostasis and fibrinolysis such as PLG, carrier proteins such as SERPINA6, hormones such as IGF2, prohormones such as AGT and SERPINA7, and apolipoproteins such as APOA1. This number of proteins may suggest that the proteins which are produced in the liver and secreted into the blood have a high percentage of secretion in liver-specific genes.

We further investigated the disease status of the 317 liver-specific genes by querying for diseases of the liver (Figure 1d). We found that 248 (77.3%) out of the 317 liver-specific genes are associated with liver-related diseases. For example, those liver-related diseases include MESH:D006394 (Hemangiosarcoma), MESH:D006501(Hepatic Encephalopathy), MESH:D006527 (Hepatolenticular Degeneration), MESH:D008103 (Liver Cirrhosis), MESH:D008107 (Liver Diseases), and MESH:D010382 (Peliosis Hepatis). 245 (99%) out of the 248 are validated as directly related to the liver by Disease Ontology [15]. We, therefore, concluded that liver-specific genes/proteins identified by HOMER are more likely to be associated with diseases related to the liver. In the future, we will test whether this conclusion can be applied to the other organs.

### Case study 2: organ-specific gene set enrichment analysis

We downloaded microarray data from GEO [20] for six organs: lung, ovary, prostate, bladder, pancreas, and kidney (Table 3). We then created a phenotype table of normal and disease states for each reference series. Next, we built 52 organ-specific gene sets (for example, a lung-specific gene set consists of 115 organ-specific genes, an ovary-specific gene set 96 organ-specific genes, a prostate-specific gene set 144 organ-specific genes, a bladder-specific gene set 71 organ-specific genes, a pancreas-specific gene set 161 organ-specific genes, and a kidney-specific gene set 191 organ-specific genes) and 10 random non-specific gene sets using the organ-specific gene set enrichment analysis method explained in the method section.

**Table 3:** Statistics of GEO microarray data for GSEA

Organ	Disease	#Samples	Reference series
Lung	Lung-sarcoidosis	12	GSE16538
Lung	adenocarcinoma(Lung Tumor)	107	GSE10072
Lung	Cystic Fibrosis	20	GSE2395
Lung	Squamous Lung Cancer	10	GSE3268
Lung	Malignant pleural mesothelioma	54	GSE2549
Lung	Lung-Cancer	192	GSE4115
Prostate	prostate cancer	104	GSE6099
Prostate	metastatic prostate tumor	6	GSE7930
Prostate	metastatic prostate tumor	164	GSE6919
Prostate	prostate tumors	30	GSE3868
Ovary	ovarian cancer	24	GSE14407
Ovary	Serous Carcinoma	37	GSE10971
Ovary	polycystic ovary syndrome	15	GSE5090
Ovary	Ovarian Endometriosis	20	GSE7305
Bladder	carcinomas	60	GSE3167
Bladder	Urothelial carcinoma	17	GSE24152
Pancreas	soft tissue sarcoma	39	GSE2719
Pancreas	multistep pancreatic carcinogenesis	22	GSE19650
pancreas	Pancreatic Ductal Adenocarcinoma	78	GSE15471
pancreas	Clinic Pancreatic Tumor	52	GSE16515
Kidney	renal cell carcinoma	20	GSE6344
Kidney	hyperaldosteronism	15	GSE8514
Kidney	preeclampsia	6	GSE6573
Kidney	metastatic prostate tumors	164	GSE6919

**Figure 4:**
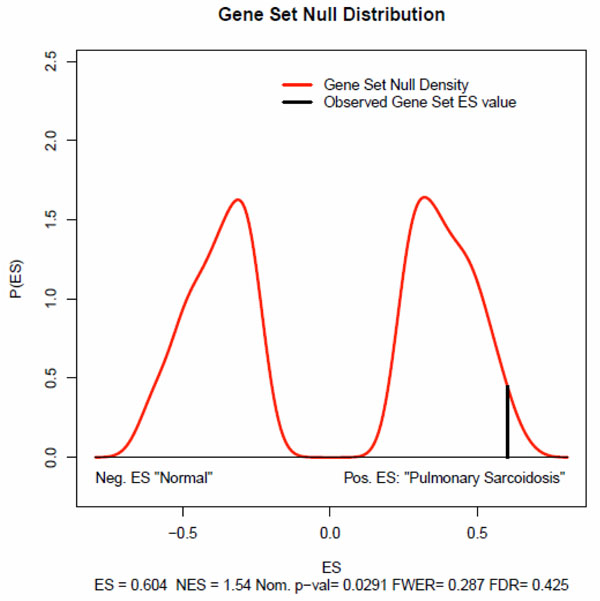
**GSEA tesults of GSE16538 for lung-specific gene set.** A null distribution for the ES was generated based on permuted phenotype labels and recomputed ES of the gene set for the permutated data. The empirical, nominal p-value of the observed ES is then calculated relative to this null distribution.

After preparing the three data files -- expression datasets, phenotype labels, and gene sets-- we loaded them into R-GSEA, set the analysis parameters, and ran the analysis for every reference series. For example, the GSEA results for GSE16538 are shown in Figure 4. The genome-wide gene expression profiles in GSE16538 were compared in tissues derived from subjects with active pulmonary sarcoidosis (n=6) and those with normal lung anatomy (n=6). Its original purpose was to test the hypothesis that tissue genome-wide gene expression analysis, coupled with gene network analyses of differentially expressed genes, would provide novel insights into the pathogenesis of pulmonary sarcoidosis [21].

For the lung-specific gene set, five key statistics for the gene set enrichment analysis were reported, Enrichment Score (ES) (0.604), Normalized Enrichment Score (NES)(1.54), familywise-error rate (FWER)(0.287), False Discovery Rate (FDR)(0.425), and Nominal P Value(0.0291). The normalized enrichment score (NES) is the primary statistic for examining gene set enrichment results [22]. By normalizing the enrichment score, GSEA accounts for differences in gene set size and in correlations between gene sets and the expression dataset; therefore, we used the normalized enrichment scores (NES) to compare analysis results across organ-specific gene sets and non-organ-specific gene sets.

**Figure 5:**
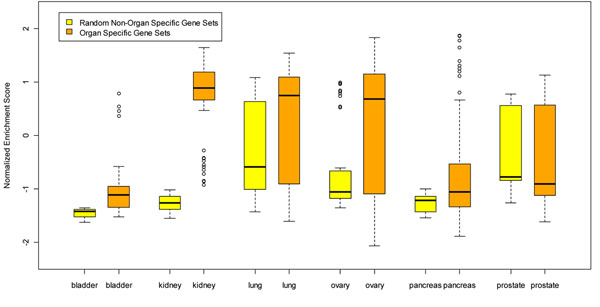
**Organ-specific gene sets analysis for lung, ovary, prostate, bladder, pancreas, and kidney.** The median normalized enrichment scores of organ-specific gene sets are markedly higher than that of random non organ-specific gene sets, in lung, ovary, bladder, pancreas, and kidney, except for prostate.

Figure 5 displays the normalized enrichment score for all 52 organ-specific gene sets and 10 random non-organ-specific gene sets over the six organs: lung, ovary, prostate, bladder, pancreas, and kidney. We can see that in the bladder, kidney, lung, ovary and pancreas, the medians of the normalized enrichment scores for organ-specific gene sets are above those of the random non-specific gene sets. This might suggest that organ-specific gene sets are more likely to become enriched in disease samples. On the other hand, we didn’t see this characteristic in the prostate. In the prostate, the normalized enrichment scores for organ-specific gene sets are very similar to random non-specific gene sets. Validation for more organs is planned in the future to test our hypothesis that organ specificity of a gene/protein correlates with associated diseases.

## Conclusion

We developed HOMER as an integrated database system to query, analyze, and characterize organ-specific genes/proteins. HOMER integrates many different types of organ-specific molecular information: organ-specific genes/proteins from the dbEST [13], TiSGED [11], and HPA [12] databases; disease gene relationship from the CTD [14] database; and disease organ relationships from the Disease Ontology [15] database. Organ-specific genes/proteins can be searched, displayed, and downloaded from our online user interface. The current HOMER database can help users address a wide range of organ specificity related questions in human disease studies. We also developed a statistical method for organ-specific genes/proteins, which can be extended to other organisms. Last, our database was evaluated by comparison to other known databases and two case studies.

## Discussion

In this paper, we have demonstrated that HOMER can be used to discover and characterize organ-specific genes/proteins from experimental data and to test the hypothesis that there is correlation between the organ specificity of a gene/protein and the associated diseases. In Case Study 1, we showed that liver-specific genes/proteins identified by HOMER are more likely to be associated with diseases related to the liver. And in case study 2, we showed that organ-specific gene sets are more likely to become enriched in disease samples in the lung, ovary, bladder, pancreas, and kidney, but not in the prostate. It is obvious that more data and analysis, validation methods and tools, and clinical trials are needed to translate organ-specific biomarkers to clinical applications. With ongoing efforts and as more disease and microarray data are collected, HOMER can become a useful resource to investigate the relationship between organ specificity and organ-related disease.

In biology, an organ is a group of tissues that perform a specific function or group of functions. There are 4 primary tissue types in the human body: epithelial tissue, connective tissue, muscle tissue and nerve tissue. And there are 12 major organ systems in the human body: Circulatory System, Lymphatic System, Digestive System, Endocrine System, Integumentary System, Muscular System, Nervous System, Reproductive System, Respiratory System, Skeletal System, Urinary/Excretory Systems, and Embryonic System. Usually there is a main tissue and sporadic tissues in an organ. For example, the heart is mostly composed of fibroblasts and to some extent of cardiomyoc[1,24,25]. Based on the main tissue and the human organ system, we categorized the tissues in dbEST into organs. We found some tissues difficult to categorize in this way, for example, adipose tissue, peritoneum and leiomios (leiomyoma). Since there are too many libraries of those tissues in the dbEST, we decided to categorize them into separate organs with the same name of the tissues.

Adipose tissue and peritoneum don't really belong to any organ system. Adipose tissue is more commonly known as fat, and it helps cushion the skin and provide protection from cold temperatures. All the peritoneum really does is lubricate and drain the abdomen. A leiomyoma (leiomios) is a benign smooth muscle neoplasm that is not premalignant. It can occur in any organ, but the most common forms occur in the uterus, small bowel and the esophagus. In the dbEST, there are 58 libraries which list leiomios, an uncharacterized tissue, as an organ, for example in lib.3508 (http://www.ncbi.nlm.nih.gov/nucest/20967784).

There are also several potential limitations to this study. First, some libraries in dbEST are not labeled clearly for tissues or organs. For example, in lib.50 to lib.70, we cannot get any information about tissues or organs. Second, there are 44 libraries in dbEST which are mixed, such as Lib.589, which pools human melanocyte, fetal heart, and pregnant uterus. We removed these before data analysis. The last possible limitation to the study relates to the relatively small or even absence of microarray sample numbers in some organs. For example, most organs have only 2 to 5 reference series which contain normal and disease states, and there is no microarray data with both normal and disease states for amnion, blood vessel, bone, ear, embryo, gallbladder, ganglia, leiomios, rectum, salivary gland, spinal cord, spleen, thymus, tonsil, trachea, umbilical cord, and ureter. However, with the ongoing development of HOMER and GEO [20], more microarray data will become available and be collected, and more organ-specific genes/proteins may be validated.

## Methods

### Pathway analysis, gene ontology categorization, and drug target analysis of organ-specific genes/proteins

We used pathway analysis, gene ontology analysis and drug target analysis to unravel the intricate pathways, functional contexts and targeting drug, and this approach is essential to the understanding of molecular mechanisms of organ-specific genes/proteins.

### Function annotation analysis

DAVID database was used to study biological process in gene ontology. Fisher's exact test is used to test the statistical significance for association between the gene list with expression changes and the function set [26].

### Pathway-gene association matrix

Pathway comparisons were performed using the following databases: Kyoto Encyclopedia of Genes and Genomes (http://www.genome.ad.jp/kegg/) [27] and HPD [28]. The visualization for the pathway-gene association matrix was created by Excel 2010 VBA.

### Drug-target analysis

Drugs and drug targets were retrieved from Drugbank [29]. A light-weight implementation of the Document Object Model interface in Python 2.7.l [30], xml.dom.minidom, was used to parse the XML format data.

### Data source

**Figure 6:**
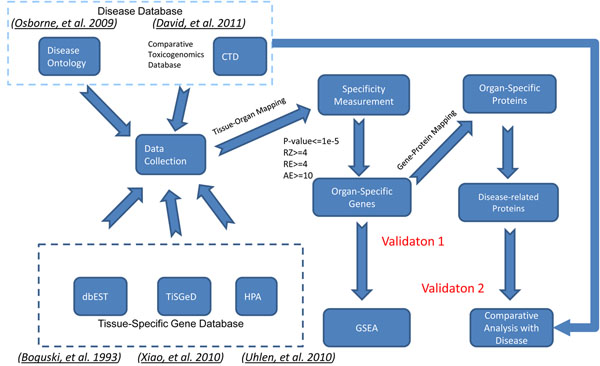
**Data integration process.** The whole data integration process was divided into three steps: 1) organ-specific biomarker colletion from dbEST, TiSGeD, and HPA; 2) disease data collection from CTD and disease ontology; and 3) validation: 3a)gene set enrichment analysis and 3b) disease comparative analysis.

We show an overview of the data integration process in Figure 6. Organ-Specific Markers data in HOMER were collected from three different sources, i.e., dbEST [13], TiSGeD [11], and HPA [12].

Raw data of EST reports from dbEST (at 04/19/2011) were downloaded from NCBI. We retrieved the “dbEST ID”, “EST name”, “GenBank Acc”, “Lib Name”, “Tissue type”, and “Organ” for each EST library under condition that the “Organism” in the EST library is Homo sapiens.

Based on "Lib Name", "Tissue Type", and "Organ", each library was categorized into a corresponding organ category, according to the TissuDB tissue hierarchy [45], tissue-type terms and their ontological hierarchy in Tissue Ontology [31], and disease of anatomical entity in Disease Ontology [15]. Briefly, our library categorization process is described as follows. For libraries with a definite “Organ”, we categorized by “Organ”. For libraries with no “Organ”, we referred to the descriptions of “Lib Name” and “Tissue Type” and their hierarchy in TissuDB [45], Tissue Ontology [31], or Disease Ontology [15] and manually categorized them into a corresponding organ. Libraries without a definite pathological description were removed. Last, organs with gene number less than 100 and EST number less than 300 were excluded. In all, we downloaded 8,314,483 human ESTs from 8,723 EST libraries, and the screening process described above left us with 8,031 libraries and 6,351,056 ESTs distributed in 111,367 UniGene IDs after converting from “GenBank Acc” and 52 organs (Table 4).

**Table 4:** dbEST Statistics for human organs

# of ESTs	# of Libraries	# of UniGenes	ORGAN	Human Organ System
1343245	990	41075	brain	Nervous System
525690	293	37481	testis	Reproductive System
453069	416	33210	lung	Respiratory System
391891	127	26930	liver	Digestive System
259386	74	26809	eye	Nervous System
324056	258	26629	uterus	Reproductive System
261443	202	25571	kidney	Excretory System
347930	385	23174	placenta	Embryonic System
280145	45	22673	embryo	Embryonic System
202761	27	22367	spleen	Immune System
279494	986	22261	colon	Digestive System
369626	1140	21953	breast	Reproductive System
288058	93	21901	skin	Integumentary System
339113	350	21370	prostate	Reproductive System
252414	48	20678	pancreas	Digestive System
154320	72	20072	bone	Skeletal System
148343	49	19875	heart	Circulatory System
142041	42	17744	muscle	Muscular System
188269	354	17319	stomach	Digestive System
132396	194	16936	ovary	Reproductive System
172902	139	16801	blood	Circulatory System
108962	29	16781	lymph node	Lymphatic System
100250	35	14619	blood vessel	Circulatory System
92800	47	13154	thymus	Immune System
109850	329	13012	bone marrow	Immune System
85617	58	12465	nerve	Nervous System
92072	267	11901	mouth	Digestive System
82442	401	11618	thyroid	Endocrine System
48816	35	10716	small intestine	Digestive System
105486	37	9622	cervix	Reproductive System
41381	30	9500	adrenal gland	Endocrine System
55430	6	9278	trachea	Respiratory System
56134	168	8413	pharynx	Respiratory System
36857	66	8310	bladder	Excretory system
56676	9	7428	lymph	Lymphatic System
47340	273	7392	larynx	Respiratory System
23085	4	7005	parathyroid gland	Endocrine System
22976	15	6229	pituitary	Endocrine System
21731	22	5906	esophagus	Digestive System
16066	19	5764	adipose	
19241	6	5470	ear	Nervous System
23920	10	5146	salivary gland	Digestive System
9612	7	4296	ganglia	Nervous System
20474	13	3719	tonsil	Immune System
14660	17	3502	umbilical cord	Embryonic System
5555	2	2816	ureter	Excretory System
12127	63	2749	amnion	Embryonic System
6327	4	2420	rectum	Digestive System
10640	58	2218	leiomios	
2853	8	1566	gallbladder	Digestive System
2346	3	1368	spinal cord	Nervous System
366	7	195	peritoneum	

TiSGeD [11] is a database consisting of genes with an associated SPM, which is a measure of its tissue specificity. SPM values range from 0 to 1.0. Currently there are 2423 human genes from 107 tissues from different organs which have an SPM value above 0.9. A user can also retrieve the data of organ-specific genes, which will be a collection of different tissues constituting that organ. Thus, for the organs of our interest, we include the organ-specific genes having SPM values >0.9.

In HPA, we have 4,842 proteins and their expressions across 48 tissues. The expression data were obtained based on analysis of immunohistochemistry-based images in [32] and categorized as negative/weak/moderate/strong. HPA also provides a list of 74 proteins which are found to be expressed in only one cell type.

**Figure 7:**
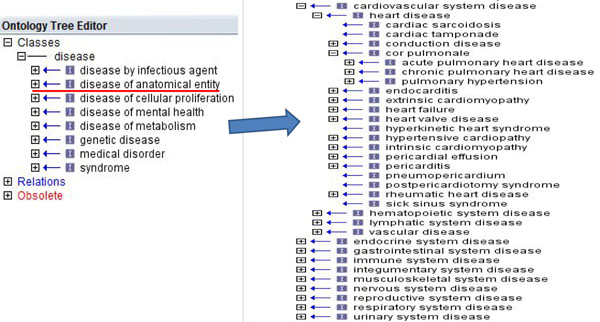
**Disease ontology.** The class of ‘disease of anatomical entity’ in the left panel contains 10 subclasses in the right panel. Each subclasses can be further expanded into sub-subclasses.

The Comparative Toxicogenomics Database CTD[14] and Disease Ontology [15] were used to extract the associations between disease and gene/protein and between organ and disease, respectively. We first used perl to convert the Disease Ontology file in OBO format to a relational table in tab-delimited format. Then we used OBO-Edit [33] to open the Disease Ontology file in OBO format and manually parsed the association for each disease and each organ in the disease of anatomical entity (Figure 7). For example, we categorized 25 diseases into the breast (Table 5). After the two steps of parsing, the disease and organ relationships contain 46 organs and 7,850 diseases, 2,600 of which can be mapped into MeSH ID.

**Table 5:** 25 primary diseases related to breast

ChildID	ChildName	ChildMSHID	ParentID	ParentName	ParentMSHID	Organ
			DOID:10349	solitary cyst of breast		breast
DOID:10349	solitary cyst of breast		DOID:10350	breast cyst	MESH:D047688	breast
DOID:10686	lactocele		DOID:10350	breast cyst	MESH:D047688	breast
			DOID:10351	mammary duct ectasia		breast
			DOID:10352	fibroadenosis of breast		breast
			DOID:10353	fibrosclerosis of breast		breast
DOID:3274	proliferative type fibrocystic change of breast		DOID:10354	breast fibrocystic disease	MESH:D005348	breast
DOID:5997	non-proliferative fibrocystic change of the breast		DOID:10354	breast fibrocystic disease	MESH:D005348	breast
			DOID:10686	lactocele		breast
DOID:12698	gynecomastia	MESH:D006177	DOID:10688	hypertrophy of breast		breast
DOID:13520	neonatal infective mastitis		DOID:10690	mastitis	MESH:D008413	breast
			DOID:10691	fat necrosis of breast		breast
			DOID:11603	infant gynecomastia	MESH:D006177	breast
			DOID:12698	gynecomastia	MESH:D006177	breast
			DOID:13520	neonatal infective mastitis		breast
DOID:3463	breast disease	MESH:D001941	DOID:15	reproductive system disease		breast
DOID:12698	gynecomastia	MESH:D006177	DOID:1923	sex differentiation disease	MESH:D012734	breast
DOID:8310	sclerosing adenosis of breast		DOID:3274	proliferative type fibrocystic change of breast		breast
DOID:10350	breast cyst	MESH:D047688	DOID:3463	breast disease	MESH:D001941	breast
DOID:10688	hypertrophy of breast		DOID:3463	breast disease	MESH:D001941	breast
DOID:10690	mastitis	MESH:D008413	DOID:3463	breast disease	MESH:D001941	breast
DOID:10691	fat necrosis of breast		DOID:3463	breast disease	MESH:D001941	breast
DOID:11603	infant gynecomastia	MESH:D006177	DOID:3463	breast disease	MESH:D001941	breast
DOID:5998	microglandular adenosis	MESH:D005348	DOID:3463	breast disease	MESH:D001941	breast
DOID:9504	benign mammary dysplasia		DOID:3463	breast disease	MESH:D001941	breast
			DOID:5996	blunt duct adenosis of breast		breast
DOID:10353	fibrosclerosis of breast		DOID:5997	non-proliferative fibrocystic change of the breast		breast
DOID:5996	blunt duct adenosis of breast		DOID:5997	non-proliferative fibrocystic change of the breast		breast
DOID:5999	apocrine adenosis of breast		DOID:5997	non-proliferative fibrocystic change of the breast		breast
DOID:5996	blunt duct adenosis of breast		DOID:5998	microglandular adenosis	MESH:D005348	breast
DOID:5999	apocrine adenosis of breast		DOID:5998	microglandular adenosis	MESH:D005348	breast
DOID:7312	breast adenomyoepithelial adenosis		DOID:5998	microglandular adenosis	MESH:D005348	breast
DOID:8310	sclerosing adenosis of breast		DOID:5998	microglandular adenosis	MESH:D005348	breast
DOID:8335	microglandular adenosis of breast		DOID:5998	microglandular adenosis	MESH:D005348	breast
			DOID:5999	apocrine adenosis of breast		breast
			DOID:7312	breast adenomyoepithelial adenosis		breast
			DOID:8310	sclerosing adenosis of breast		breast
			DOID:8335	microglandular adenosis of breast		breast
DOID:10349	solitary cyst of breast		DOID:9504	benign mammary dysplasia		breast
DOID:10351	mammary duct ectasia		DOID:9504	benign mammary dysplasia		breast
DOID:10352	fibroadenosis of breast		DOID:9504	benign mammary dysplasia		breast
DOID:10354	breast fibrocystic disease	MESH:D005348	DOID:9504	benign mammary dysplasia		breast

The Gene–Disease Relationships were downloaded from CTD [14] website in CSV format and contained 20,444 genes and 4,290 diseases as of April 7, 2011, 1,096 of which were in common with the diseases in the disease and organ relationships.

The microarray datasets and their latest gene chip annotation files were derived from NCBI GEO [20]. Phenotype tables for each reference series were manually created based on the description of samples we downloaded.

### Statistics

We developed a statistic model based on p-value, z-score and number of ESTs to determine organ specificity of genes.

Given *p* to be the probability of success in a Bernoulli trial where one EST in gene *i* falls in organ *j*, the probability of *x* successes is

Where *K* is the total number of ESTs in gene *i*, *M* is the total number of ESTs in organ *j*, *N* is the total number of ESTs in Human, *p*=*M* / *N*, and *x* is the number of ESTs corresponding to gene *i* in organ *j*.

The p-value for gene *i* in organ *j* is the probability of obtaining a test statistic at least as extreme as the one observed, given that the null hypothesis that there is no enrichment between gene *i* and organ *j* is true, and calculated according to the formula

The absolute expression value (*AE*, or #EST) of gene *i* in organ *j* is defined as *x*, the number of ESTs corresponding to gene *i* in organ *j*. The expected expression value (*EE*) of gene *i* in organ *j* is defined as the expected number of ESTs of gene *i* in organ *j* under the null hypothesis that the two variables, gene and organ, are independent of each other.

The relative expression value (*RE*, or Adjusted #EST) of a gene *i* in organ *j* is defined as *AE*/*EE*.

The absolute z-score (*AZ*) shown as follows is used to indicate how many standard deviations an observed absolute expression value in gene *i* above the mean

Similarly, the relative z-score (*RZ*) is calculated by

We define the genes as organ-specific genes if they satisfy the four criteria (i.e. p-value ≤10^–5^, *RZ* ≥ 4, *RE* ≥ 4, and *AE* ≥ 10). We determine the parameters based on the following four criteria: 1) *AE* must be greater than the average absolute expression value of all genes, 2) *RE* must be greater than the average relative expression value of the genes identified by criteria 1, 3) at least 95% of identified organ-specific genes are absolute organ-specific gene, and 4) the more organ-specific genes identified, the better.

If a gene is identified as specific to one organ, it is called single-organ-specific gene or absolute organ-specific gene. On the other hand, if a gene is identified as specific to multiple organs, it is called multiple-organ-specific gene or relative organ-specific gene.

First, we set *AE* ≥ 10 according to experience after rounding to 10 the mean absolute expression value of all the genes in our database, which is 9.56.

Second, we set *RZ* ≥ 4 according to experience after rounding to 4 the mean relative expression value of all the rest genes in our database after filtering with the first criteria, which is 3.85.

Suppose z-score be from a standard normal distribution, one-tailed p-value of testing the hypothesis that there is no enrichment between gene *i* and organ *j* is

For example, p-value is equal to 2.28E-02, 1.35E-03, 3.17E-05, 2.87E-07, and 9.87E-10, respecitively, when z-score is equal to 2, 3, 4, 5, and 6. We round the p-values and obtain five pairs: (p-value≤10^–2^, *RZ* ≥ 2), (p-value≤10^–3^, *RZ* ≥ 3), (p-value≤10^–5^, *RZ* ≥ 4), (p-value≤10^–7^, *RZ* ≥ 5 ), and (p-value≤10^–10^, *RZ* ≥ 6).

Comparison of the four pairs of parameters is shown in Table 6. The threshold (p-value≤10^–10^, *RZ* ≥ 6) is too strict. It filters out about two third of the organ-specific genes that are identified by (p-value≤10^–2^, *RZ* ≥ 2). The thresholds (p-value≤10^–2^, *RZ* ≥ 2) and (p-value≤10^–3^, *RZ* ≥ 3) cannot satisfy the second criteria that requires at least 95% of identified organ-specific genes are absolute organ-specific gene. Finally, we choose (p-value≤10^–5^, *RZ* ≥ 4) as thresholds based on the forth criteria because we can identify more organ-specific genes with (p-value≤10^–5^, *RZ* ≥ 4) than with (p-value≤10^–7^, *RZ* ≥ 5).

**Table 6:** A comparison of four pairs of P-value and Z-score thresholds

	#OSG	#R-OSG	#A-OSG	%A-OSG
p-value ≤ 10^–2^, *RZ* ≥ 2	9597	1913	7684	80%
p-value ≤ 10^–3^, *RZ* ≥ 3	8434	923	7511	89%
p-value ≤ 10^–5^, *RZ* ≥ 4	6569	168	6401	97%
p-value ≤ 10^–7^, *RZ* ≥ 5	4622	0	4622	100%
p-value ≤ 10^–10^, *RZ* ≥ 6	2903	0	2903	100%

### Organ-specific gene set enrichment analysis

Our method for organ-specific gene set enrichment analysis includes three steps: 1) collecting microarray data from GEO [20] and creating phenotype tables for each reference series, 2) producing organ-specific gene sets, and 3) running R-GSEA in R programming environment and performing statistical analysis. R-GSEA is the R version of the GSEA program [22]. In order to run it, R release 2.0 or later is required.

We downloaded microarray expression data from GEO [20] for six organs: bladder, kidney, lung, ovary, pancreas, and prostate. The datasets must have data on normal and diseased state with respect to the six organ, based on which we created phenotype tables. We then built an organ-specific gene sets for each of 52 organs. For the comparison of our organ-specific gene set, we built 10 non-specific gene sets by randomly picking up genes which were sufficiently lower ranked to the organ or specific to other organs. We compared the organ-specific gene set(s) with the non-specific gene sets to determine if the organ-specific gene set was significantly enriched, while other gene sets were not being enriched with regards to a diseased state related to that organ.

### Online HOMER server design

The online version of HOMER database is a typical 3-tier web application, with an Oracle10g database at the backend database service layer, Apache/PHP server scripts to the middleware application web server layer, and CSS-driven web pages presented on the browser.

**Figure 8:**
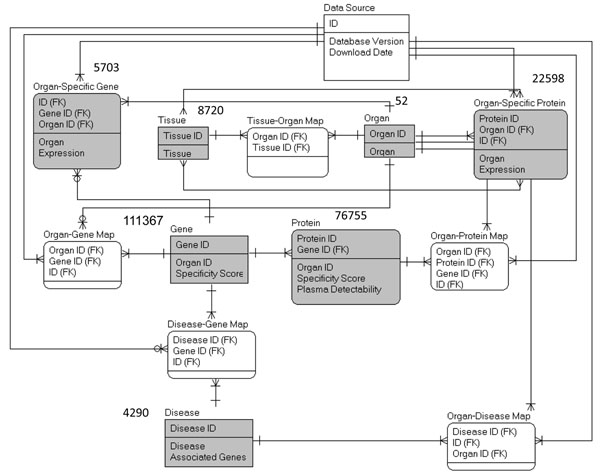
**Relational metadata model.** The datasets derived by the data generation pipeline are filled in gray.

The result tables derived from the data generation steps were imported into the Oracle10g database (Figure 8). The organ-gene, disease-gene, organ-disease, organ-protein, and tissue-organ mapping tables enable users to query the database with different IDs.

## Authors' contributions

JYC conceived the initial work and designed the method for the database construction. FZ generated the datasets, developed the statistics method, the database backend and the web-based interface, and performed the statistical analyses of the case studies. All authors are involved in the drafting and revisions of the manuscript.

## Competing interests

The authors declare that they have no competing financial interests.

## Supplementary Material

Additional File 1 **154 Organ-specific genes.** AE: absolute expression RE: relative expression RZ: relative z-score Click here for file

Additional File 2 **The pathway-gene association matrix of 154 organ-specific genes.** In the organ-gene association matrix, 1 stands for presence of a gene in a pathway and 0 for absence. Click here for file

Additional File 3 Drug target analysis of 154 organ-specific genes. Click here for file
