# Supplementary material for: HOMER: a human organ-specific molecular electronic repository
Source: BMC Bioinformatics. 2011 Oct 18;12(Suppl 10):S4. doi: 10.1186/1471-2105-12-S10-S4 (PMC3236847; doi:10.1186/1471-2105-12-S10-S4)
Supplement: Additional File 1 — 154 Organ-specific genes. AE: absolute expression RE: relative expression RZ: relative z-score [file 1471-2105-12-S10-S4-S1.doc]

**Additional File 1 - 154 Organ-specific Genes**

| **Unigene ID** | **Gene Name** | **Organ** | **AE** | **p-value** | **RE** | **RZ** | **Validation by HPA** |
| --- | --- | --- | --- | --- | --- | --- | --- |
| Hs.558396 | SCD | adipose | 103 | 4.51E-88 | 17.2 | 6.71 | No |
| Hs.50813 | ITLN1 | adipose | 40 | 6.32E-74 | 145.65 | 6.66 | No |
| Hs.4 | ADH1B | adipose | 59 | 3.69E-66 | 31.21 | 6.19 | No |
| Hs.184927 | CYP11B1 | adrenal gland | 103 | 0 | 168.96 | 7.07 | No |
| Hs.438016 | CYP17A1 | adrenal gland | 723 | 0 | 176.08 | 7.07 | No |
| Hs.654479 | CYP21A2 | adrenal gland | 568 | 0 | 196.07 | 7.07 | No |
| Hs.374596 | TPT1 | amnion | 242 | 0 | 29.13 | 6.82 | No |
| Hs.203717 | FN1 | amnion | 208 | 0 | 26.31 | 6.49 | Yes |
| Hs.644065 | TSC22D2 | amnion | 45 | 8.24E-82 | 147.37 | 7.06 | No |
| Hs.53985 | GP2 | bladder | 199 | 0 | 82.04 | 6.96 | No |
| Hs.654951 | DPM1 | bladder | 88 | 0 | 33.94 | 6.56 | No |
| Hs.437060 | CYCS | bladder | 229 | 0 | 23.56 | 5.39 | No |
| Hs.99855 | FPR2 | blood | 114 | 0 | 34.37 | 7.05 | No |
| Hs.846 | CXCR2 | blood | 142 | 0 | 35.63 | 7.04 | No |
| Hs.194778 | CXCR1 | blood | 187 | 0 | 34.11 | 6.98 | No |
| Hs.59736 | KRT28 | blood vessel | 81 | 0 | 67.67 | 7.07 | No |
| Hs.449625 | GRM8 | blood vessel | 85 | 0 | 51.49 | 7.07 | No |
| Hs.80962 | NTS | blood vessel | 185 | 0 | 51.13 | 7.06 | No |
| Hs.375129 | MMP3 | bone | 1482 | 0 | 44.17 | 7.07 | No |
| Hs.2159 | ACAN | bone | 152 | 0 | 33.82 | 6.97 | No |
| Hs.605153 | TCF4 | bone | 557 | 0 | 18.53 | 6.92 | No |
| Hs.380781 | DEFA1 | bone marrow | 214 | 0 | 76.92 | 7.07 | No |
| Hs.458272 | MPO | bone marrow | 303 | 0 | 63.06 | 7.06 | Yes |
| Hs.710507 | LILRA1 | bone marrow | 109 | 0 | 68.36 | 7.06 | No |
| Hs.176977 | OLIG2 | brain | 154 | 0 | 5.39 | 7.05 | Yes |
| Hs.590575 | GRM3 | brain | 614 | 0 | 5.49 | 7.05 | No |
| Hs.517729 | MLC1 | brain | 1855 | 0 | 5.34 | 7.04 | No |
| Hs.516153 | SV2A | brain | 1051 | 0 | 4.74 | 6.66 | Yes |
| Hs.353022 | ETAA1 | breast | 116 | 0 | 19.81 | 6.73 | No |
| Hs.485158 | SPDEF | breast | 275 | 0 | 21.76 | 6.71 | No |
| Hs.462946 | FBXL20 | breast | 142 | 0 | 14.79 | 6.14 | No |
| Hs.160786 | ASS1 | cervix | 118 | 0 | 16.57 | 6.58 | No |
| Hs.2785 | KRT17 | cervix | 321 | 0 | 21.69 | 4.5 | No |
| Hs.186350 | RPL4 | cervix | 103 | 1.28E-39 | 5.11 | 4.33 | No |
| Hs.592227 | NOX1 | colon | 75 | 0 | 32.85 | 7.06 | No |
| Hs.489355 | MUC12 | colon | 486 | 0 | 30.02 | 6.98 | No |
| Hs.315 | MUC2 | colon | 160 | 0 | 28.92 | 6.47 | No |
| Hs.21016 | COCH | ear | 171 | 0 | 206.98 | 7.07 | No |
| Hs.518726 | IBSP | ear | 67 | 0 | 283 | 7.07 | No |
| Hs.644125 | KCTD12 | ear | 114 | 0 | 73.61 | 7.06 | No |
| Hs.533779 | CLDN6 | embryo | 131 | 0 | 18.04 | 6.51 | No |
| Hs.300141 | RPL39 | embryo | 425 | 0 | 8.92 | 5.57 | No |
| Hs.156367 | RPS29 | embryo | 437 | 0 | 6.48 | 4 | No |
| Hs.412484 | OLR1 | esophagus | 64 | 0 | 91.49 | 7.06 | No |
| Hs.242057 | CRNN | esophagus | 71 | 0 | 204.79 | 7.03 | Yes |
| Hs.654550 | KRT13 | esophagus | 711 | 0 | 146.49 | 6.89 | Yes |
| Hs.307096 | LACRT | eye | 82 | 0 | 27.98 | 7.07 | No |
| Hs.66739 | KRT12 | eye | 340 | 0 | 28.75 | 7.07 | No |
| Hs.46275 | CRYBA1 | eye | 209 | 0 | 28.06 | 7.07 | No |
| Hs.76452 | CRP | gallbladder | 50 | 0 | 468.75 | 7.07 | No |
| Hs.300774 | FGB | gallbladder | 62 | 0 | 120.73 | 7.01 | No |
| Hs.713625 | FGG | gallbladder | 66 | 0 | 86.59 | 6.92 | No |
| Hs.654404 | HLA-B | ganglia | 62 | 1.25E-42 | 11.14 | 5.77 | No |
| Hs.591486 | MPZ | ganglia | 19 | 4.25E-34 | 125.24 | 7.01 | No |
| Hs.77961 | HLA-B | ganglia | 44 | 2.13E-25 | 8.18 | 5.11 | No |
| Hs.75636 | MYL7 | heart | 352 | 0 | 44.01 | 7.07 | Yes |
| Hs.75640 | NPPA | heart | 115 | 0 | 34.22 | 7.03 | No |
| Hs.533613 | TNNT2 | heart | 143 | 0 | 36.38 | 7 | Yes |
| Hs.654425 | UMOD | kidney | 2328 | 0 | 29.37 | 7.07 | Yes |
| Hs.369252 | SLC22A6 | kidney | 88 | 0 | 22.93 | 7.06 | No |
| Hs.527830 | KCNJ1 | kidney | 78 | 0 | 28.26 | 7.05 | Yes |
| Hs.531111 | YLPM1 | larynx | 136 | 0 | 58.43 | 7.05 | No |
| Hs.475348 | BEND5 | larynx | 96 | 0 | 120.54 | 7.05 | No |
| Hs.443735 | SETD8 | larynx | 82 | 0 | 63.24 | 7.05 | No |
| Hs.712713 | ST13 | leiomios | 328 | 0 | 215.93 | 7.07 | No |
| Hs.584927 | YY1AP1 | leiomios | 277 | 0 | 273.27 | 7.07 | No |
| Hs.632466 | CTSK | leiomios | 64 | 0 | 87.54 | 7.05 | No |
| Hs.654443 | C9 | liver | 98 | 0 | 20.75 | 7.07 | No |
| Hs.1219 | ADH4 | liver | 173 | 0 | 18.67 | 6.9 | Yes |
| Hs.12907 | CYP2E1 | liver | 174 | 0 | 16.51 | 6.89 | Yes |
| Hs.1074 | SFTPC | lung | 819 | 0 | 16.17 | 7.05 | No |
| Hs.523084 | SFTPA1 | lung | 176 | 0 | 14.76 | 7.05 | Yes |
| Hs.535295 | SFTPA1 | lung | 293 | 0 | 14.02 | 7.04 | No |
| Hs.657869 | CPNE5 | lymph | 74 | 0 | 57.63 | 6.95 | No |
| Hs.2484 | TCL1A | lymph | 199 | 0 | 79.98 | 5.6 | No |
| Hs.535192 | EEF1A1 | lymph | 1439 | 0 | 6.31 | 4.69 | No |
| Hs.479384 | SEL1L3 | lymph node | 135 | 0 | 19.78 | 6.87 | No |
| Hs.262886 | INPP5D | lymph node | 189 | 0 | 33.9 | 6.54 | Yes |
| Hs.513870 | ATP2A3 | lymph node | 119 | 0 | 24.25 | 6.52 | No |
| Hs.372914 | NDRG1 | mouth | 743 | 0 | 19.82 | 6.82 | No |
| Hs.379821 | FAM83A | mouth | 99 | 0 | 48.87 | 6.73 | No |
| Hs.504115 | TRIM29 | mouth | 237 | 0 | 31 | 6.56 | No |
| Hs.181768 | MYOD1 | muscle | 76 | 0 | 53.32 | 7.07 | No |
| Hs.523403 | TNNI2 | muscle | 201 | 0 | 50.06 | 7.05 | Yes |
| Hs.709681 | TMOD4 | muscle | 106 | 0 | 46.91 | 7.04 | Yes |
| Hs.485278 | RNF8 | nerve | 107 | 0 | 23.4 | 7.02 | No |
| Hs.37044 | PRPH | nerve | 73 | 0 | 49.68 | 6.74 | No |
| Hs.407604 | MED14 | nerve | 124 | 0 | 26.86 | 6.6 | No |
| Hs.1154 | OVGP1 | ovary | 188 | 0 | 43.59 | 7.01 | No |
| Hs.314359 | EIF3K | ovary | 310 | 0 | 15.76 | 6.03 | No |
| Hs.111903 | FCGRT | ovary | 230 | 0 | 11.54 | 5.5 | No |
| Hs.632211 | CTRB2 | pancreas | 333 | 0 | 28.46 | 7.07 | No |
| Hs.449281 | PRSS1 | pancreas | 3464 | 0 | 28.33 | 7.07 | No |
| Hs.46835 | IAPP | pancreas | 325 | 0 | 28.44 | 7.07 | Yes |
| Hs.37045 | PTH | parathyroid gland | 88 | 0 | 310.2 | 7.07 | Yes |
| Hs.435615 | CASR | parathyroid gland | 31 | 9.71E-67 | 216.18 | 6.84 | Yes |
| Hs.399891 | CD109 | parathyroid gland | 45 | 4.09E-65 | 61.43 | 6.97 | Yes |
| Hs.1288 | ACTA1 | peritoneum | 18 | 4.72E-40 | 351.35 | 7.02 | Yes |
| Hs.334347 | CKM | peritoneum | 12 | 1.5E-24 | 213.73 | 6.92 | No |
| Hs.211092 | PLUNC | pharynx | 319 | 0 | 134 | 7.07 | Yes |
| Hs.432416 | TCHH | pharynx | 168 | 0 | 109.62 | 7.06 | No |
| Hs.362854 | C1QTNF9 | pharynx | 52 | 0 | 140.32 | 7.04 | No |
| Hs.655229 | GH1 | pituitary | 820 | 0 | 374.76 | 7.07 | No |
| Hs.1897 | POMC | pituitary | 380 | 0 | 344.56 | 7.07 | No |
| Hs.1905 | PRL | pituitary | 993 | 0 | 363.81 | 7.07 | No |
| Hs.654390 | CSH1 | placenta | 2387 | 0 | 22.85 | 7.07 | No |
| Hs.709192 | PSG1 | placenta | 1530 | 0 | 22.42 | 7.07 | No |
| Hs.654413 | PSG3 | placenta | 1007 | 0 | 22.63 | 7.07 | No |
| Hs.537218 | SEMG2 | prostate | 854 | 0 | 34.09 | 7.07 | No |
| Hs.1968 | SEMG1 | prostate | 357 | 0 | 33.03 | 7.06 | No |
| Hs.255462 | MSMB | prostate | 632 | 0 | 30.8 | 7.04 | No |
| Hs.516105 | ACTG2 | rectum | 202 | 0 | 250.69 | 7.02 | No |
| Hs.436568 | CD74 | rectum | 224 | 0 | 30.43 | 4.18 | No |
| Hs.520640 | ACTB | rectum | 159 | 2.18E-78 | 7.03 | 4.8 | No |
| Hs.123114 | CST1 | salivary gland | 195 | 0 | 233.45 | 7.07 | No |
| Hs.650650 | SMR3B | salivary gland | 142 | 0 | 127.85 | 7.04 | No |
| Hs.654495 | STATH | salivary gland | 171 | 0 | 102.54 | 7.01 | No |
| Hs.99476 | SERPINA12 | skin | 125 | 0 | 24.29 | 7.06 | No |
| Hs.601077 | C1orf68 | skin | 133 | 0 | 24.89 | 7.06 | No |
| Hs.654510 | FLG | skin | 94 | 0 | 21.95 | 7.04 | Yes |
| Hs.655233 | DEFA5 | small intestine | 84 | 0 | 123.17 | 7.07 | No |
| Hs.521459 | ADAMDEC1 | small intestine | 207 | 0 | 106.87 | 7 | No |
| Hs.643431 | IGJ | small intestine | 230 | 0 | 41.53 | 5.81 | No |
| Hs.1787 | PLP1 | spinal cord | 32 | 4.71E-27 | 15.49 | 6.62 | No |
| Hs.433670 | FTL | spinal cord | 35 | 7.27E-27 | 12.91 | 5.38 | No |
| Hs.134602 | TTN | spinal cord | 19 | 8.97E-27 | 54.66 | 5.98 | No |
| Hs.2200 | PRF1 | spleen | 102 | 0 | 22.87 | 6.75 | Yes |
| Hs.712539 | HBG1 | spleen | 936 | 0 | 19.38 | 6.4 | No |
| Hs.449630 | HBA1 | spleen | 624 | 0 | 15.84 | 6.28 | No |
| Hs.69319 | GKN1 | stomach | 262 | 0 | 38.5 | 7.07 | No |
| Hs.523130 | LIPF | stomach | 756 | 0 | 42.15 | 7.07 | No |
| Hs.512714 | WNT7B | stomach | 197 | 0 | 29.05 | 6.85 | No |
| Hs.120831 | C16orf82 | testis | 144 | 0 | 13.25 | 7.07 | No |
| Hs.127510 | KLHL10 | testis | 107 | 0 | 13.27 | 7.07 | No |
| Hs.120319 | NT5C1B | testis | 104 | 0 | 14.13 | 7.07 | No |
| Hs.386698 | C10orf27 | thymus | 113 | 0 | 67.55 | 7.07 | No |
| Hs.1309 | CD1A | thymus | 90 | 0 | 68.81 | 7.07 | No |
| Hs.1310 | CD1B | thymus | 156 | 0 | 76.1 | 7.07 | No |
| Hs.654591 | TG | thyroid | 1585 | 0 | 132.07 | 7.07 | Yes |
| Hs.469728 | PAX8 | thyroid | 127 | 0 | 55.62 | 6.99 | No |
| Hs.520898 | CTSB | thyroid | 234 | 0 | 9.62 | 5.87 | No |
| Hs.419463 | RPL23A | tonsil | 237 | 0 | 21.37 | 6.81 | No |
| Hs.98910 | RFTN1 | tonsil | 75 | 0 | 54.62 | 6.68 | No |
| Hs.546269 | RPL10A | tonsil | 137 | 0 | 16.73 | 6.58 | No |
| Hs.234742 | LPO | trachea | 62 | 0 | 96.21 | 7.04 | No |
| Hs.529517 | LTF | trachea | 2813 | 0 | 86.15 | 7.04 | No |
| Hs.614734 | PROM1 | trachea | 96 | 0 | 38.43 | 7.02 | No |
| Hs.524121 | ROBO4 | umbilical cord | 189 | 0 | 170.98 | 7.07 | No |
| Hs.471851 | HDLBP | umbilical cord | 144 | 0 | 17.59 | 6.82 | No |
| Hs.517033 | TGM2 | umbilical cord | 194 | 0 | 28.02 | 6.75 | No |
| Hs.387804 | PABPC1 | ureter | 39 | 4.6E-29 | 12.39 | 6.31 | No |
| Hs.450230 | IGFBP3 | ureter | 12 | 6.32E-08 | 8.03 | 4.73 | No |
| Hs.198241 | AOC3 | uterus | 185 | 0 | 11.2 | 5.71 | No |
| Hs.173716 | ADAM33 | uterus | 103 | 8.6E-93 | 13.9 | 5.43 | No |
| Hs.532325 | PAEP | uterus | 79 | 3.79E-60 | 10.78 | 5.1 | No |
